# Cruelty to Animals

**Published:** 1867-03

**Authors:** 


### Editorial Department.

### Cruelty to Animals.

There has been recently, an active effort in Buffalo, to stimulate movement to prevent cruelty to animals, aud clergymen in some of the churches have preached discourses upon the inhumanity of cruelly treating the brute creation. We have no very positive data, but we believe this movement is in secret sympathy with the New York Society, whose President, Mr. Bergh, has figured so largely in communications to the Medical Colleges, protesting against vivisections in teaching physiology, also appearing before the public in descriptions of physiological experiments, and the general methods of teaching by experiments upon living animals. It is quite astonishing what torrents of sympathy are poured out in behalf of a few cats, dogs and rabbits, which are experimented upon by physiologists with the view of more clearly impressing upon young men who are about to assume the responsible duties of the medical profession, some of the more central truths of the philosophy of life. It would hardly appear probable that any reasonable being who expects to render an account of himself at the judgment, would now join Mr. Bergh in his crusade upon medical teaching, and especially would this be true, if he has read the reply of Professor John C. Dalton both to Mr. Bergh, and before the New York Academy of Medicine, which is so complete and conclusive as to leave nothing more to be said. After refuting most completely the objections urged against vivisection; that it is cruel, that it is liable to uncertainty, that it has not led to valuable result, Dr. Dalton proceeds to enumerate some of the discoveries to which it has led; first, to the discovery of the circulation of the blood; second, to many important facts concerning respiration; third, to transfusion of the blood; fourth, to artificial respiration; fifth, to much of what is known of digestion—that the stomach secretes the gastric juice, and does it only during digestion, and when the stomach is excited, and that it has the power of dissolving the food—is a true digestive secretion; sixth, a great many facts concerning the functions of the nervous system; seventh, the modern operation for aneurism; eighth, improvements in the surgical operation of exsection and extirpation, by discovery of the office and functions of the periosteum in the regeneration of bone; ninth, in the treatment of bites by venomous serpents, and much of what we know of the anatomy of the fangs, poison-glands and accessory parts in the rattlesnake—the mode of production and discharge of the poison, its composition and properties, its action on various species and classes of animals, and the value of different remedies and antidotes; tenth, a knowledge of the origin and prevention of parasitic disease.

Whoever reads Dr. Dalton's paper, will never more speak of vivisections by physiologists, being cruel, uncertain or deceptive, and, above all, fruitless of valuable results.

We have nothing to say upon the legitimate question of preventing cruelty to animals; the abuse or unnecessary torture of any animal is repulsive to all the feelings of humanity; the poor, debased, drunken, sordid, soulless scoundrel, who shames the world by acts of cruelty to animals, should be abated from the sight of men, and nothing can be more unqualified than our condemnation of brutes who are guilty of such crimes. When this just sense of decency for the care and protection of the animal creation, is perverted into a foolish, inconsistent crusade against gentlemen of the highest culture, with the purest motive, and the most unselfish effort for human good, then, it is, that we protest against their action, and begin to look upon them as bigoted, superstitious, self-righteous usurpers, who would fain make the world believe that goodness and kindness and humanity will die with them.

This whole subject is very likely to be presented to the public in all its various bearings and in full detail; there is an evident pressure from outside, or our ministers to Christian churches would not have felt called upon to preach gospel sermons upon the subject of cruelty to animals. In Buffalo we do not think that animals are treated badly to any great extent, at least; the dogs are treated as well as many of the women, and the cats, rabbits and other domestic animals, including rats and mice, are general favorites with a large portion of the inhabitants. The horses are not abused by their owners, that we have ever observed in a single instance, and the cows of Buffalo are not confined in ill-ventilated stables or forced to give milk in greater quantity, or of purer quality, than consistent with their general good keeping. We have recently heard of some children who were put in beer-casks instead of pantaloons, and were not fed on the choicest food, which comes nearest to being within the scope of the text, of anything we have ever known. If the morality and christianity of Western New York, has arrived to so great a degree of perfection, that its Reverend advocates can legitimately espouse the cause of the brute creation by direct appeal, or more properly, if decency and common humanity have so completely died out of the hearts of the church congregations, as to make it necessary to preach against "cruelty to animals," in their Sabbath or other gatherings, then we would respectfully suggest, that future efforts for christianizing be abandoned, and our refined circles of church-going citizens be invited instead, to attend horse-races and gladiatorial contests in modern amphitheatres, as more productive ot kindness and humanity than the exercises which have thus far proved of so little avail.

This sympathy for animals, at least the New York exhibition of it, as understood by an outsider, is a hypocritical, nonsensical flustration about nothing, and in Buffalo we believe it is only an echo of the same confusion.

